# Does an alar ligament tubercle exist? Anatomical study with application to imaging interpretation and skull base approaches

**DOI:** 10.1007/s10143-026-04489-2

**Published:** 2026-09-18

**Authors:** Nilo Alvarez Toledo, Carmine Antonio Donofrio, Filippo Badaloni, Antonio Fioravanti, David Ezra, Noritaka Komune, Rizwan Aslam, Joseph Lockwood, Joe Iwanaga, Aaron S. Dumont, Marios Loukas, R. Shane Tubbs

**Affiliations:** 1 Department of Neurosurgery, Icahn School of Medicine at Mount Sinai, New York, NY USA; 2 Department of Neurosurgery, ASST Cremona, Cremona, Italy; 3 Department of Neurosurgery, IRCCS Istituto Delle Scienze Neurologiche di Bologna, Bologna, Italy; 4 School of Nursing Sciences, The Academic College of Tel Aviv Jaffo, Tel Aviv Jaffo, Israel; 5 Department of Biological Anthropology, Natural History Museum, Cleveland, OH USA; 6 Department of Otorhinolaryngology, Graduate School of Medical Sciences, Kyushu University, Fukuoka, Japan; 7 Department of Otolaryngology, Tulane University School of Medicine, New Orleans, LA USA; 8 Department of Neurosurgery, Tulane University School of Medicine, New Orleans, LA USA; 9 Department of Neurosurgery and Ochsner Neuroscience Institute, Ochsner Health System, New Orleans, LA USA; 10 Department of Anatomical Sciences, St. George’s University, St. George’s, Grenada; 11 Department of Surgery, Tulane University School of Medicine, New Orleans, LA USA; 12 University of Queensland, Brisbane, Australia; 13 Department of Neurosurgery, University of Virginia, Charlottesville, VA USA; 14 Department of Neurosurgery, Tulane University School of Medicine, 131 S. Robertson St., Suite 1300, New Orleans, LA 70112 USA

**Keywords:** Anatomy, Surgery, Occipital condyle, Alar ligament, Craniocervical junction, Posterior cranial fossa, Far-lateral transcondylar approach

## Abstract

Although numerous anatomical and radiological studies describe a “tubercle” of the alar ligaments at the medial OC as the alar ligament insertion, no systematic osteological investigation has evaluated the prevalence, morphology, and variability of this structure. Therefore, this study aimed to determine whether an alar ligament tubercle (ALT) is a consistent anatomical feature or a morphological variant. Two hundred fifty adult skulls were examined. The presence, morphology, and laterality of the ALTs were documented. Tubercle dimensions were measured using microcalipers, and a classification system was developed to detail their anatomy. High-resolution inferior-view photographs were used to outline the OCs and corresponding ALTs, and ImageJ was used to calculate the proportion of OC surface area occupied by the ALT. An ALT was identified in eight (3.2%), occurring bilaterally in seven specimens and unilaterally in one. Three morphological types were observed: small (≤ 3 mm), large (> 3 mm), and irregular/spicule-like forms. The tubercles occasionally extended to the anterior foramen magnum margin. The mean ALT dimensions were length 4.1 mm, width 3.4 mm, and thickness 4.0 mm. Five tubercles were classified as type 1 (33.3%), four as type 2 (26.7%), and six as type 3 (40.0%). The ALT occupied 10–24% (mean 15%) of the OC surface area. The ALT is a rare anatomical variant rather than a consistent OC landmark. Awareness of this variation could be useful for accurate radiological interpretation, avoidance of diagnostic pitfalls, and safe planning of skull base approaches involving the OC.

## Introduction

The occipital bone is divided into basilar (basioccipital), squamous, and two lateral condylar parts [1] The lateral condylar portions, located on each side of the foramen magnum (FM), bear the occipital condyles (OCs) on their inferior surfaces, which articulate with the superior articular facets of the atlas (C1) [1]. The OCs are paired prominences lateral to the FM that converge in a posterolateral-to-anteromedial orientation. The alar ligaments, along with the transverse ligament, provide most of the stability of the craniovertebral junction (CVJ) [2–5]. They attach medially to the lateral surface of the odontoid process of the axis and extend laterally and cranially on the medial portion of the OCs and/or on the anterolateral FM aspect [3, 6].

**Fig. 1:**
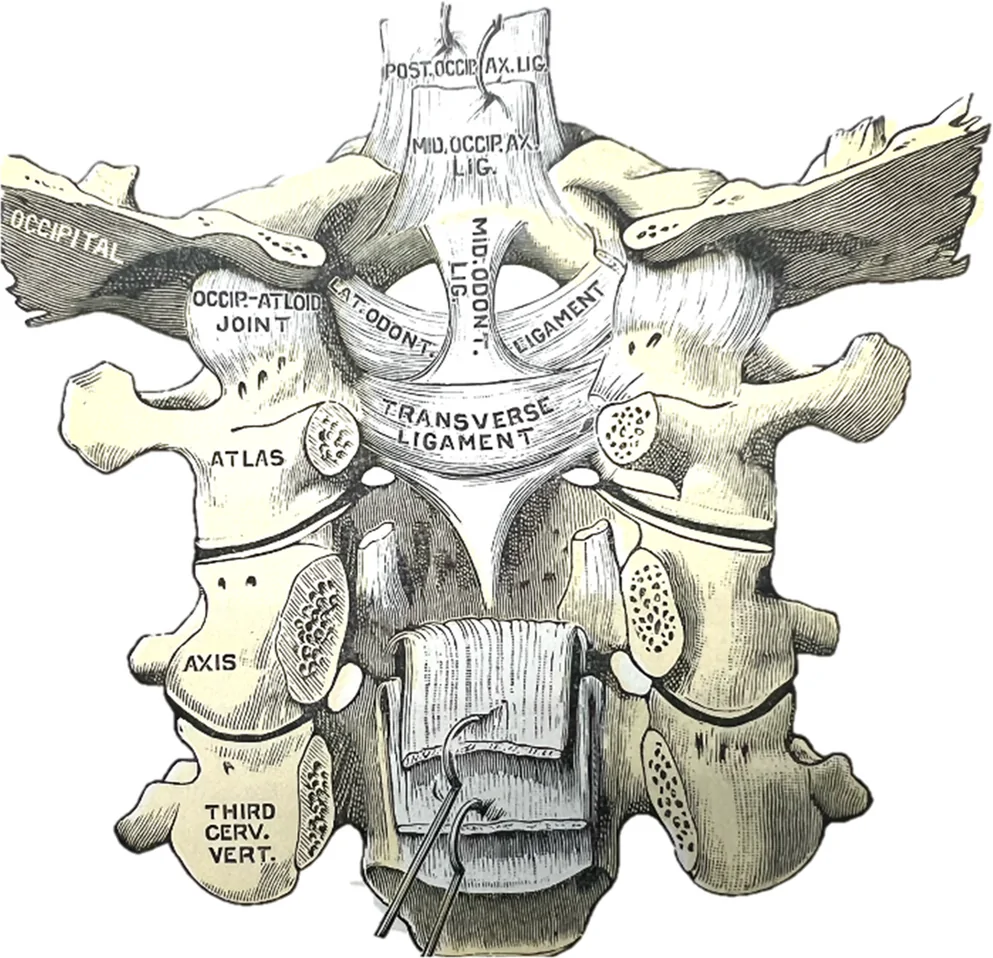
Posterior view of the ligaments of the craniocervical junction, noting the position of the alar ligaments (Lat. Odont. Ligament) (after [17])

High-quality anatomical studies of the CVJ consistently describe a distinct bony prominence on the medial surface of each OC, termed the “alar ligament tubercle” (ALT), as the site of alar ligament attachment (Fig. 1) ([7–13]). Rhoton’s microsurgical study of the FM [14] describes “a tubercle on the medial side of each condyle for attachment of the alar ligament,” emphasizing its importance as a surgical landmark. On the basis of detailed anterior FM dissections, de Oliveira et al. [15] similarly reported that “a tubercle situated on the medial aspect of each occipital condyle gives attachment to the alar ligament of the odontoid process.” Offiah and Day further note that “on the medial aspect of the occipital condyle, a small tubercle marks the bony attachment of the alar ligament,” confirming its anatomical and radiological identification [16]. The study published by Jhawar and colleagues corroborates these findings, identifying “an alar tubercle on the medial surface of the condyles” as the characteristic site of alar ligament insertion in both cadaveric specimens and imaging correlations [10]. Collectively, these studies affirm the existence, location, and surgical relevance of the ALT on the medial aspect of the OC.

However, to our knowledge, no published studies have characterized the prevalence, morphology, or variation of the ALT. Because the alar ligaments are critical for CVJ stability and can be injured in various pathologies, such as whiplash injury [18], the objective of the present anatomical study is to elucidate the prevalence, characteristics, and anatomical variations of the ALT.

## Methods

Two hundred fifty adult dry skulls were examined for ALTs. One hundred and fifty of them were from the Hamann-Todd skeletal collection housed at the Cleveland Museum of Natural History (Cleveland, OH, USA), and the remaining were from the teaching collection at Tulane University School of Medicine (New Orleans, LA, USA). The 150 skulls from the Hamann-Todd skeletal collection had known ethnicity, sex, and age, while the others, aside from being adults, did not. The age range for the first cohort, comprising 81 males and 69 females, was 18 to 95 years (mean, 55 years).

The prevalence and exact location of ALT were documented. Width, length, and thickness were measured three times, and the mean values were recorded all by the same observer (RST). All measurements were made using microcalipers (Mitutoyo, Kawasaki, Japan).

ALTs were classified into three types: type 1 (≤ 3 mm in width), type 2 (> 3 mm in width), and type 3 (with irregular morphology).

High-resolution inferior-view photographs centered on the FM were obtained. The areas of each OC and associated ALT were manually outlined and quantified using ImageJ v1.54f (NIH, Bethesda, MD, USA). The Polygon ROI Tool was used to trace the outer boundary of the OC following the dark outline. A second ROI was traced around the ALT region, corresponding to the blue-filled area. ImageJ was used to calculate the area of each ROI in pixels². The percentage of the OC surface area occupied by the ALT was calculated as follows:

$$\:{Percent\:Tubercle\:Area}=\left(\frac{{A}_{{tubercle}}}{{A}_{{condyle}}}\right)\times\:100$$

All measurements were obtained twice for each OC and ALT, and the mean values were used for analysis. Descriptive statistics (mean, range, and standard deviation) were calculated using GraphPad Prism 10 (GraphPad Software, Boston, MA, USA). Left and right ALT dimensions were compared using a paired Student’s *t* test (or Wilcoxon signed-rank test if non-normally distributed). Statistical significance was defined as *p* < 0.05.

All internationally accepted guidelines for cadaveric donor studies were followed throughout the present study [19–23].

## Results

**Fig. 2:**
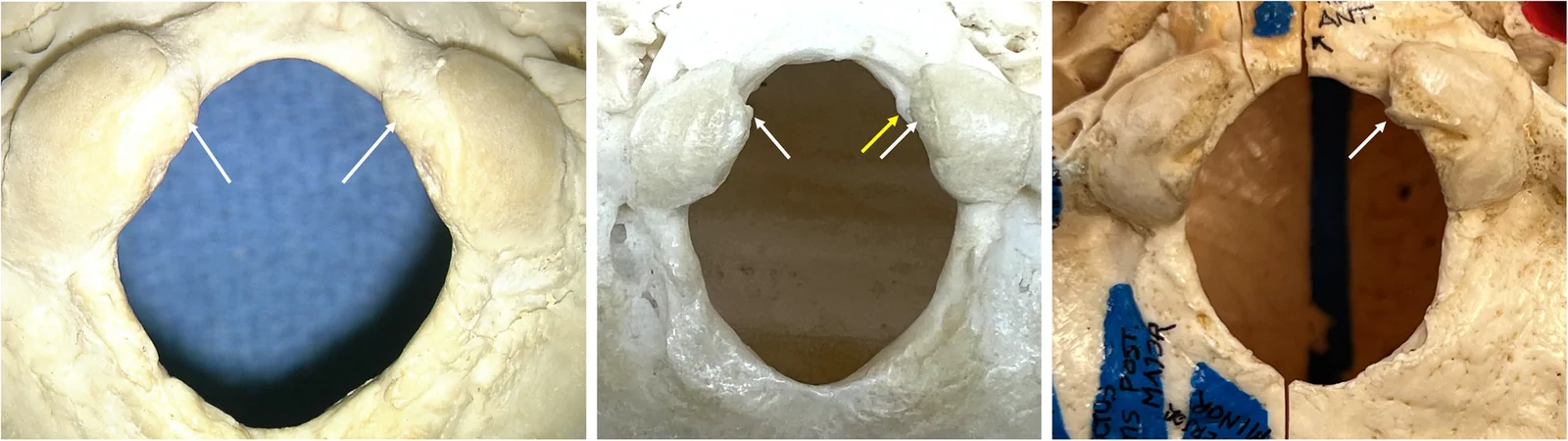
Three examples of type 1 alar ligament tubercles (white arrows). Note the additional adjacent tubercle (yellow arrow) on the foramen magnum for the middle image, and that for the right image, no tubercle is shown on the right side

**Fig. 3:**
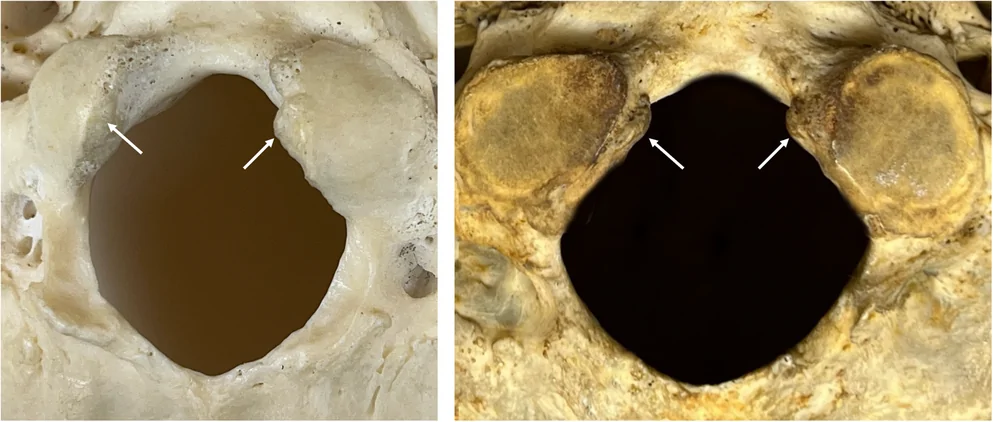
Two examples of type 2 alar ligament tubercles (white arrows)

**Fig. 4:**
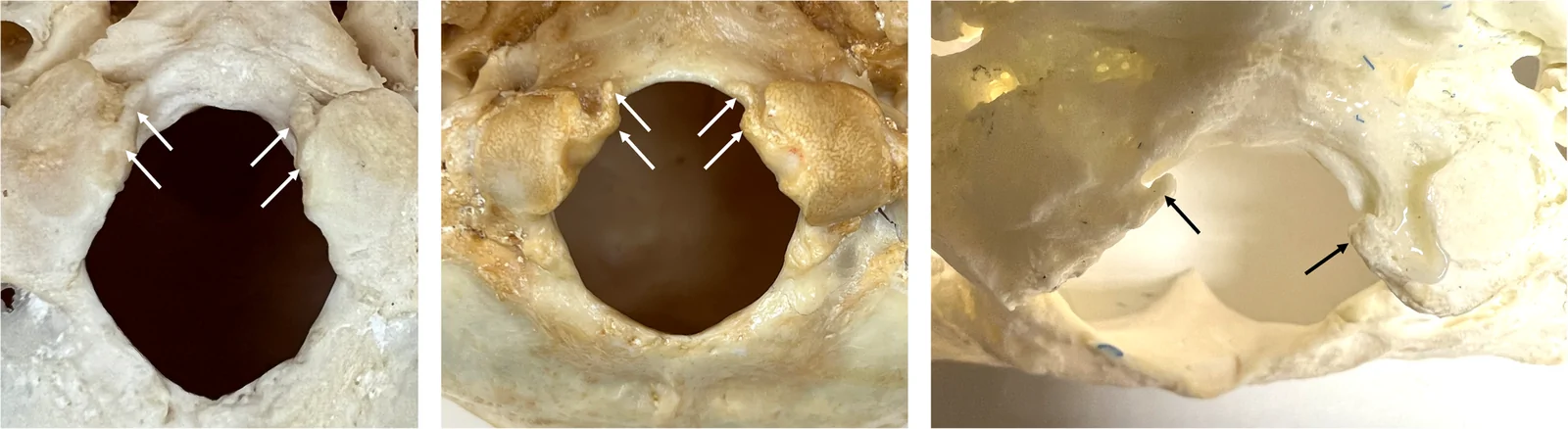
Three examples of type 3 alar ligament tubercles (white and black arrows)

An ALT was identified in eight of the 250 skulls (3.2%) (Figs. 2, 3 and 4). ALTs were bilateral in all but one of these eight skulls (Fig. 2). They were usually tubercular in shape (Figs. 2 and 3), but three were spicule-like. In two spicule-like ALT cases, part of the spiculae extended to the anterior edge of the FM (Fig. 4).

Five ALTs (33.3%) were classified as type 1 (Fig. 2), four (26.7%) as type 2 (Fig. 3), and six (40.0%) as type 3 (Fig. 4). ALT types were not always symmetrical between left and right sides (Fig. 3).

The mean length of the ALTs was 4.1 mm (range: 2.5–5.2 mm; SD = ± 0.7 mm), the mean width was 3.4 mm (range: 3.0–4.5 mm; SD = ± 0.4 mm), and the mean thickness was 4 mm (range: 3.0–5.0 mm; SD = ± 0.5 mm). The dimensions of the left and right ALTs did not differ significantly. No statistically significant differences were identified between the left and right ALT length (*p* = 0.74), width (*p* = 0.58), or thickness (*p* = 0.81).

The mean percentage of the OC surface area occupied by the ALT was 15% (range: 10–24%).

## Discussion

### Imaging considerations

The ALT, identified in 3.2% of specimens, should be regarded as an anatomical variant rather than a normal, consistent OC’s feature. Previous reports suggesting the ALT as typical [24, 25] are not supported by our findings. Therefore, the practical value of the knowledge gained from this study is that an ALT should not be expected as part of routine imaging of the CVJ.

**Fig. 5:**
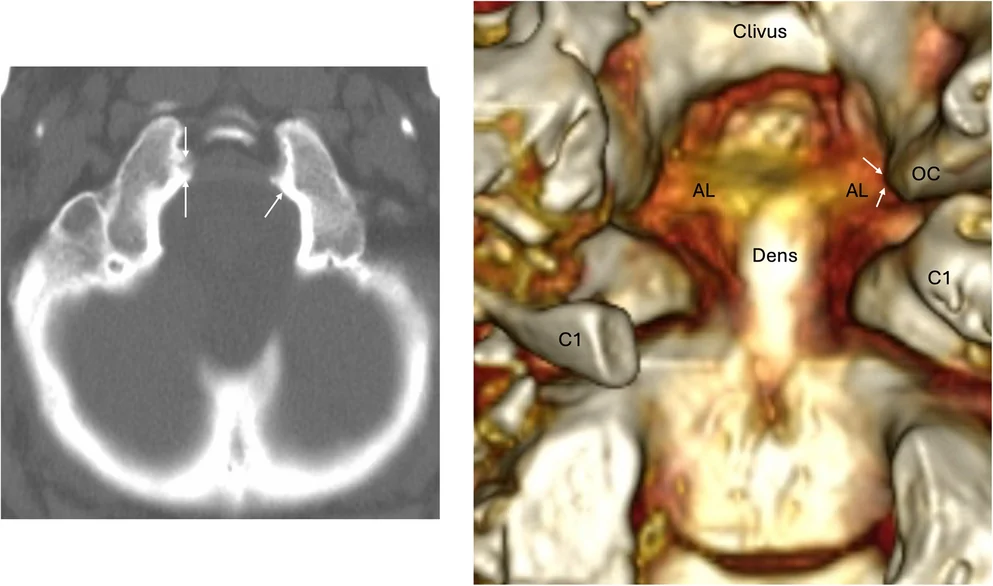
Two examples of imaging of the alar ligament tubercles. Left: Axial CT through the occipital condyles and noting left and right alar ligament tubercles (arrows). Right: 3D reconstruction (posterior view) of the cranial cervical junction and noting the left and right alar ligaments (AL) attaching onto the occipital condyles (OC). Note the alar ligament tubercle on the right side (arrows). C1 = atlas

This structure should not be misinterpreted on imaging as ossification of the alar ligaments or as an avulsion of the OC [26] (Fig. 5). Imaging artifacts may further complicate evaluation of the alar ligament’s attachment to the OC. For example, Bloom et al., [27] described nodular calcification of the alar ligament that simulated a fracture. Based on our study, such a calcification might represent the ALT. Therefore, although the ALT was present in only a minority of the skulls examined in this study, awareness of its existence is important in patients with suspected CVJ injuries.

### Surgical considerations

The far-lateral transcondylar approach, first described by Seeger [28], provides enhanced exposure of the anterolateral brainstem [29]. In these procedures, the attachment of the alar ligament to the medial aspect of the OC must be carefully considered, as its transection may compromise CVJ stability [30, 31].

Transcondylar screw fixation carries a risk of medial OC fracture [32], which may be mistaken on imaging for, or encompass, the ALT when present.

**Fig. 6:**
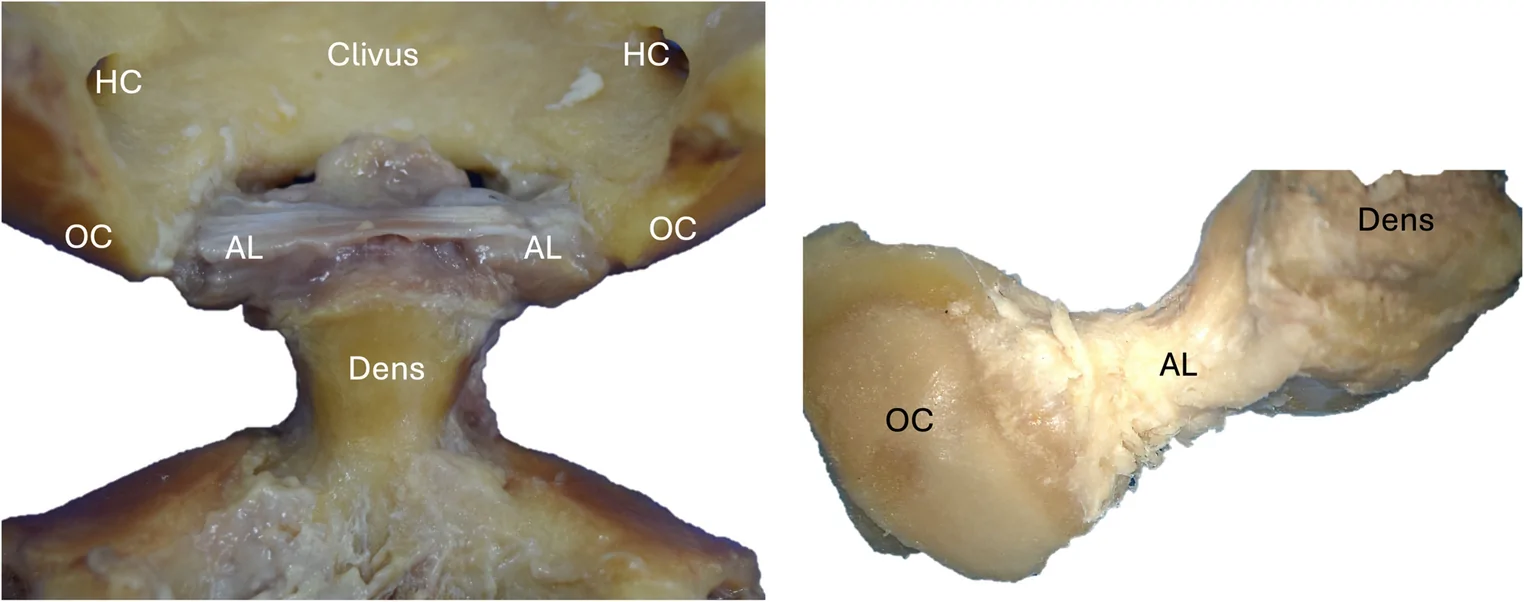
Cadaveric dissections of the alar ligaments and their attachment onto the occipital condyles. Left: Posterior view noting the occipital condyles (OC), alar ligaments (AL), and hypoglossal canals (HC). Right: removed right occipital condyle (OC) showing its connection to the alar ligament (AL). Medially, the dens is seen from an inferior view after its disconnection from the remaining body of the C2 vertebra

Type 3 °C fractures, the most common, occur near the ligament’s attachment to the medial OC and are unstable because of the OC avulsion [27, 33] (Fig. 6). High-energy trauma, such as motor vehicle collisions, is frequently associated with alar ligament injuries.

Endoscopic endonasal and transoral odontoidectomy procedures might also require careful consideration of alar ligament anatomy, as transection or removal of the alar ligament may lead to ALT avulsion, potentially damaging the OC [34]. During odontoidectomy, the dens is drilled away from its apex superiorly to about the synchondrosis inferiorly. This leaves the alar and apical ligaments disconnected medially [35]. During such procedures, a prominent ALT might encroach on the dissection region, necessitating additional lateral drilling. In such a case, the operator would need to avoid damaging the occipital condyle.

### Variant anatomy

Newton and Potts provided a rather nonspecific description of the cranial attachment of the alar ligaments, noting only slight “bony roughenings” on the medial aspect of the OCs [36]. These features were not considered in our study due to a lack of objective identification criteria, therefore limiting interobserver reproducibility.

This study provides the first systematic definition of the ALT, quantifies its contribution to the OC surface area, and proposes a simple, reproducible classification. This framework may serve as a foundation for future investigations to validate our findings and assess the prevalence of this variant across diverse populations.

One specimen exhibited a tubercle on the FM adjacent to the medial aspect of the OC (Fig. 2), likely representing an attachment site for accessory alar ligament fibers, as described by Sardi et al., [13]. Spicule-like extensions of the ALT along the FM may also correspond to additional alar ligament attachments (Fig. 4). Interestingly, in cats, accessory fibers of the alar ligaments often attach to the capsule of the lateral atlanto-occipital joint [37].

## Limitations

As with any dry bone study, the absence of soft tissues (e.g., the alar ligament) complicates the findings, as the overlying tissues and their relationships to the bone are unknown. Additionally, 100 of our specimens had an unknown ethnicity, sex, and age, although they were known to be from adult donors [38]. As the medical history of our cohort was unknown, a history of trauma to the area studied could not be ruled out. Bony roughenings, osteophytes, and degenerative changes also could not be ruled out in these skulls. However, the tubercles identified in our study were consistent with the location, anatomy, and previous descriptions of the alar ligament tubercles. The clinical and surgical implications of this anatomical structure now need patient evidence in order to substantiate the importance of the ALT. 

## Conclusions

The ALT is observed in 3.2% of adult skulls and exhibits considerable morphological variability. When present, it contributes approximately 15% of the OC surface area. Our findings indicate that the ALT should be regarded as a rare anatomical variant rather than a consistent OC landmark.

Establishing a systematic definition of the ALT, a reproducible method to quantify its contribution to the OC surface area, and a straightforward classification system may serve as a foundation for future studies to assess the prevalence of this anatomical variant across diverse populations and validate our results. Recognition of the ALT may be clinically important, as it might prevent misinterpretation on imaging, such as alar ligament ossification or OC avulsion, and informs surgical planning for CVJ procedures. However, additional clinical and surgical studies are now necessary to validate our anatomical findings.

## Data Availability

No datasets were generated or analysed during the current study.
